# Supplementary material for: Incidence of HIV infection and associated factors among female sex workers in Côte d’Ivoire, results of the ANRS 12361 PrEP-CI study using recent infection assays
Source: PLoS One. 2022 Nov 17;17(11):e0271988. doi: 10.1371/journal.pone.0271988 (PMC9671321; doi:10.1371/journal.pone.0271988)
Supplement: S1 Data — (ZIP) [file pone.0271988.s001.zip › prep-ci-incidence-paper-main/incidence_analysis.html]

Incidence of HIV infection and associated factors among female sex workers in Côte d’Ivoire, results of the ANRS 12361 PrEP-CI study using recent infection assays


# Incidence of HIV infection and associated factors among female sex workers in Côte d’Ivoire, results of the ANRS 12361 PrEP-CI study using recent infection assays

Marcellin Nouaman & Joseph Larmarange for the ANRS 12361 Study
Group
  
2022-07-15

### Contents

- Data import &
  preparation
- Table
  1. Sociodemographic and behavioural characteristics of the surveyed FSW,
  ANRS 12361 PrEP-CI study, September 2016 - March 2017
- Table
  2. Estimated HIV incidence by HIV exposure factors among FSW, ANRS 12361
  PrEP-CI study, September 2016 – March 2017

```
library(tidyverse)
library(labelled)
library(gt)
library(gtsummary)
```

## Data import & preparation

```
d <- read_csv("analytical_dataset.csv")

d <- d %>%
  mutate(
    region =
      region %>%
        fct_relevel("San Pedro", "Abidjan"),
    condomless_for_money = 
      condomless_for_money %>%
        fct_relevel("never", "sometimes", "often / always"),
    recruitment_site =
      recruitment_site %>%
        fct_relevel("bar / “maquis”", "brothel", "hotel", "street", "other"),
    condom_use_clients = 
      condom_use_clients %>%
        fct_relevel("never / sometimes / often", "always")
  ) %>%
  set_variable_labels(
    overall = "Overall",
    region = "Study region",
    age_group = "Age group",
    education = "Highest level of education",
    nationality = "Nationality",
    years_sex_work = "How many years sex work has been practised",
    usual_price = "Usual price with clients",
    recruitment_site = "Recruitment site",
    clients_last_workday = "Number of clients during last day of sex work",
    condom_use_clients = "Condom use with clients",
    condomless_for_money = "Acceptance of condomless sexual intercourse in exchange for a large sum of money",
    sti = "Self-reported STI in the last 12 months",
    several_cities = "Practised sex work in more than one city",
    last_medical_visit = "Last medical visit with a doctor or a nurse",
    hiv_negative = "HIV rapid test was negative",
    hiv_positive = "HIV rapid test was positive",
    hiv_recent = "Recent HIV infection"
  )
```

## Table 1. Sociodemographic and behavioural characteristics of the surveyed FSW, ANRS 12361 PrEP-CI study, September 2016 - March 2017

```
d %>%
  tbl_summary(
    by = region,
    include = c(
      age_group, education, nationality, years_sex_work,
      usual_price, recruitment_site, clients_last_workday,
      condom_use_clients, condomless_for_money, sti, several_cities,
      last_medical_visit
    ),
    digits = list(all_categorical() ~ c(0, 1)),
    missing_text = "not documented"
  ) %>%
  add_overall(last = TRUE) %>%
  modify_footnote(update = everything() ~ NA) %>%
  bold_labels()
```

| **Characteristic** | **San Pedro**, N = 400 | **Abidjan**, N = 600 | **Overall**, N = 1,000 |
| --- | --- | --- | --- |
| Age group |  |  |  |
| 24 years or less | 163 (41.3%) | 280 (50.3%) | 443 (46.5%) |
| 25 years or more | 232 (58.7%) | 277 (49.7%) | 509 (53.5%) |
| not documented | 5 | 43 | 48 |
| Highest level of education |  |  |  |
| never been to school | 115 (28.9%) | 105 (17.6%) | 220 (22.1%) |
| primary | 163 (41.0%) | 219 (36.7%) | 382 (38.4%) |
| secondary / university | 120 (30.2%) | 273 (45.7%) | 393 (39.5%) |
| not documented | 2 | 3 | 5 |
| Nationality |  |  |  |
| Ivorian | 312 (78.0%) | 378 (63.0%) | 690 (69.0%) |
| other nationality | 88 (22.0%) | 222 (37.0%) | 310 (31.0%) |
| How many years sex work has been practised |  |  |  |
| 3 years or less | 250 (63.6%) | 381 (63.8%) | 631 (63.7%) |
| 4 years or more | 143 (36.4%) | 216 (36.2%) | 359 (36.3%) |
| not documented | 7 | 3 | 10 |
| Usual price with clients |  |  |  |
| 1999 FCFA (~3€) or less | 285 (71.2%) | 109 (18.2%) | 394 (39.5%) |
| 2000 FCFA (~3€) or more | 115 (28.7%) | 489 (81.8%) | 604 (60.5%) |
| not documented | 0 | 2 | 2 |
| Recruitment site |  |  |  |
| bar / “maquis” | 135 (33.8%) | 254 (42.3%) | 389 (38.9%) |
| brothel | 111 (27.8%) | 142 (23.7%) | 253 (25.3%) |
| hotel | 101 (25.2%) | 58 (9.7%) | 159 (15.9%) |
| street | 15 (3.8%) | 48 (8.0%) | 63 (6.3%) |
| other | 38 (9.5%) | 98 (16.3%) | 136 (13.6%) |
| Number of clients during last day of sex work |  |  |  |
| 1 client | 21 (5.2%) | 154 (25.8%) | 175 (17.6%) |
| 2-6 clients | 316 (79.0%) | 394 (66.0%) | 710 (71.2%) |
| 7 clients or more | 63 (15.8%) | 49 (8.2%) | 112 (11.2%) |
| not documented | 0 | 3 | 3 |
| Condom use with clients |  |  |  |
| never / sometimes / often | 78 (20.4%) | 46 (7.9%) | 124 (12.9%) |
| always | 304 (79.6%) | 533 (92.1%) | 837 (87.1%) |
| not documented | 18 | 21 | 39 |
| Acceptance of condomless sexual intercourse in exchange for a large sum of money |  |  |  |
| never | 251 (69.0%) | 513 (88.0%) | 764 (80.7%) |
| sometimes | 52 (14.3%) | 27 (4.6%) | 79 (8.3%) |
| often / always | 61 (16.8%) | 43 (7.4%) | 104 (11.0%) |
| not documented | 36 | 17 | 53 |
| Self-reported STI in the last 12 months |  |  |  |
| none | 79 (20.2%) | 262 (44.5%) | 341 (34.8%) |
| yes, at least one | 312 (79.8%) | 327 (55.5%) | 639 (65.2%) |
| not documented | 9 | 11 | 20 |
| Practised sex work in more than one city |  |  |  |
| no, 1 city only | 200 (50.3%) | 527 (88.3%) | 727 (73.1%) |
| yes, 2 cities or more | 198 (49.7%) | 70 (11.7%) | 268 (26.9%) |
| not documented | 2 | 3 | 5 |
| Last medical visit with a doctor or a nurse |  |  |  |
| less than a year | 303 (76.1%) | 367 (61.4%) | 670 (67.3%) |
| more than a year or never consulted | 95 (23.9%) | 231 (38.6%) | 326 (32.7%) |
| not documented | 2 | 2 | 4 |

## Table 2. Estimated HIV incidence by HIV exposure factors among FSW, ANRS 12361 PrEP-CI study, September 2016 – March 2017

```
calculate_incidence <- function(R, P, N, omega = .3, epsilon = .013) {
  if_else(
    R == 0,
    0,
    (R - epsilon * P) / ((1 - epsilon) * omega * N)
  )
}
```

```
generate_incidence_one_var <- function(v, data = d) {
  data$variable <- v
  data$level <- d[[v]]
  data %>%
    group_by(variable, level) %>%
    summarise(
      R = sum(hiv_recent, na.rm = TRUE),
      P = sum(hiv_positive),
      N = sum(hiv_negative),
      .groups = "drop"
    ) %>%
    mutate(incidence = calculate_incidence(R, P, N))
}


res <-
  c(
    "overall", "region", "age_group", "education", "nationality",
    "years_sex_work", "usual_price", "recruitment_site",
    "clients_last_workday", "condom_use_clients", "condomless_for_money", "sti",
    "several_cities", "last_medical_visit"
  ) %>%
  map_df(~ generate_incidence_one_var(.x)) %>%
  left_join(
    var_label(d) %>% 
      unlist() %>% 
      enframe(name = "variable", value = "variable_label"),
    by = "variable"
  ) %>%
  relocate(variable_label)
```

```
res %>% 
  select(-variable) %>%
  filter(!is.na(level)) %>%
  gt(
    groupname_col = "variable_label",
    rowname_col = "level"
  ) %>%
  fmt_number(columns = starts_with("incidence"), decimals = 1, scale_by = 100) %>%
  cols_label(incidence = "Estimated incidence per 100 person-years") %>%
  tab_style(
    style = cell_text(weight = "bold"),
    locations = list(cells_row_groups(), cells_column_labels())
  )
```

|  | R | P | N | Estimated incidence per 100 person-years |
| --- | --- | --- | --- | --- |
| Overall | | | | |
| overall population | 7 | 39 | 961 | 2.3 |
| Study region | | | | |
| San Pedro | 4 | 25 | 375 | 3.3 |
| Abidjan | 3 | 14 | 586 | 1.6 |
| Age group | | | | |
| 24 years or less | 4 | 16 | 427 | 3.0 |
| 25 years or more | 3 | 22 | 487 | 1.9 |
| Highest level of education | | | | |
| never been to school | 3 | 13 | 207 | 4.6 |
| primary | 3 | 17 | 365 | 2.6 |
| secondary / university | 1 | 9 | 384 | 0.8 |
| Nationality | | | | |
| Ivorian | 4 | 26 | 664 | 1.9 |
| other nationality | 3 | 13 | 297 | 3.2 |
| How many years sex work has been practised | | | | |
| 3 years or less | 4 | 20 | 611 | 2.1 |
| 4 years or more | 3 | 19 | 340 | 2.7 |
| Usual price with clients | | | | |
| 1999 FCFA (~3€) or less | 5 | 24 | 370 | 4.3 |
| 2000 FCFA (~3€) or more | 2 | 15 | 589 | 1.0 |
| Recruitment site | | | | |
| bar / “maquis” | 1 | 9 | 380 | 0.8 |
| brothel | 3 | 16 | 237 | 4.0 |
| hotel | 2 | 9 | 150 | 4.2 |
| street | 1 | 2 | 61 | 5.4 |
| other | 0 | 3 | 133 | 0.0 |
| Number of clients during last day of sex work | | | | |
| 1 client | 1 | 5 | 170 | 1.9 |
| 2-6 clients | 4 | 27 | 683 | 1.8 |
| 7 clients or more | 2 | 7 | 105 | 6.1 |
| Condom use with clients | | | | |
| never / sometimes / often | 3 | 9 | 115 | 8.5 |
| always | 4 | 29 | 808 | 1.5 |
| Acceptance of condomless sexual intercourse in exchange for a large sum of money | | | | |
| never | 3 | 24 | 740 | 1.2 |
| sometimes | 1 | 7 | 72 | 4.3 |
| often / always | 3 | 7 | 97 | 10.1 |
| Self-reported STI in the last 12 months | | | | |
| none | 2 | 8 | 333 | 1.9 |
| yes, at least one | 5 | 30 | 609 | 2.6 |
| Practised sex work in more than one city | | | | |
| no, 1 city only | 4 | 22 | 705 | 1.8 |
| yes, 2 cities or more | 3 | 17 | 251 | 3.7 |
| Last medical visit with a doctor or a nurse | | | | |
| less than a year | 3 | 22 | 648 | 1.4 |
| more than a year or never consulted | 4 | 17 | 309 | 4.1 |
