# Supplementary material for: Incidence of HIV infection and associated factors among female sex workers in Côte d’Ivoire, results of the ANRS 12361 PrEP-CI study using recent infection assays
Source: PLoS One. 2022 Nov 17;17(11):e0271988. doi: 10.1371/journal.pone.0271988 (PMC9671321; doi:10.1371/journal.pone.0271988)
Supplement: Attachment — Submitted filename: Response_to_reviewer_PLOS ONE_2022-04-25.docx [file pone.0271988.s002.docx]

**To the editors of the Journal of the PLOS ONE**

Abidjan, April 12^th^ 2022

**Response-to-reviewers letter and revised manuscript entitled “Incidence of HIV infection and associated factors among female sex workers in Côte d’Ivoire, results of the ANRS 12361 PrEP-CI study using recent infection assays”**

Dear Editors,

This document provides point-by-point responses to the reviewers’ comments. Their comments, reproduced in *italics*, are followed by our responses and, finally by the additional text and changes (**in bold red**) to the manuscript first submitted. We thank the reviewers for their comments which have helped improve our paper.

***Reviewer : 1***

*This is a well written paper on the factors that influence HIV positivity in Female Sex Workers from two different cities in Côte d’Ivoire. The factors that favor HIV infection are age of less than 24 years old, being non -Ivorian, less education, if the clients have paid less, larger number of clients on a single day, inconsistent use of condoms by the client, or condomless sex, especially if extra money has been paid for the same, and presence of STDs. They have also calculated the incidence of HIV infection per 100 person years and used RITA to determine recent HIV infections of less than six months .
The paper is well written and has nothing new to offer in terms of determination of factors favoring HIV infection in FSW. However RITA is the new factor mentioned in this paper.*

*Some clarifications:*

1. *Did all FSW have both rapid tests or only one as à screening test. This is not clear in line 174.*
2. *In Table 1, under duration of sex activity should it not be less than 3 years or more than 3 years. It says less than 3 years and more than 4 years. What about those between 3and 4 years. Lines 267 to 269 state only below and above 4 years. Kindly rectify.*
3. *Abidjan saw more sexual activity than San Pedro. Yet, San Pedro shows shows higher infection rates of Hiv. A sentence or two should be added to explain this in discussion.*
4. *The fact that this was a convenience sample rather than a random sample and therefore certain statistical tests could not be done should be shifted to limitations of the study. Also the fact that multivariate analysis is not available for RITA.*

**Authors’ response:**

1. All study participants were tested for HIV by two rapid tests. A clarification was made in the text as follows: « HIV screening was carried out by two rapid tests (Determine®, Alere and Vikia®, bioMérieux), for all surveyed FSWs, at the sex work sites » (line 179)
2. The remark is correct. We have harmonised in the tables and in the text as follows: « >3	years or more » Vs. « 3 years or less » (table 1, 2 and lines 280-281)
3. Yes, indeed the reviewer is right. Female sex workers (FSWs) in San Pedro were more likely to be exposed to HIV than those in Abidjan. Indeed, they charged a low price for the pass, which

results in a high number of customers. They also had a lower rate of condom use and were more likely to accept sex without condoms in exchange for a large sum of money. They also reported more STIs than those in Abidjan. All these factors favour exposure to HIV infection.

The discussion section has added some elements to this effect: « The high incidence of HIV infection further evidences this among FSWs in San Pedro compared to those practising in Abidjan. In fact, they charged a lower pass price with a higher number of customers. They also had a lower rate of condom use and were more likely to accept sex without condoms in exchange for a large sum of money, and they reported more STIs than those in Abidjan. All these factors favour exposure to HIV infection ». (lines 302-307).

4. The remark is relevant. And we have completed the limitations paragraph of the study as suggested : « Yet, we have to acknowledge some limitations. Fist, as RITA is a biological assay, classification of infections as recent or not does not rely on self-reported information. Some people with long-standing HIV infection and on treatment may be misclassified as newly infected. However, these false recent cases are taken into account, as a false recent rate is applied when estimating incidence. Secondly our data suffer from a lack of power due to a relative sample size with a few number of FSW recently HIV-infected. In addition, the comparison between San Pedro and Abidjan could not been done with statistical tests because the sample was not a random sample but rather a convenience sample». Also, we have already stated in the limitations that multivariate analyses were not available for RITA. (lines 348-356)

----------------------------------------------------------------------------------------------------------------------

***Reviewer: 2***

*This is a very interesting paper on the use of HIV Recent Infection Testing Algorithm (RITA) to measure recent HIV infection and HIV incidence in a convenience sample of FSW in two towns in Côte d'Ivoire. This study will be a great addition to the literature, especially as it informs the epidemiology of HIV among key populations. I have a few questions that I hope the authors can address to strengthen the manuscript (below):*

***Introduction***

*Can the authors explain why RITA needs to be adapted to geographic context if it is an assay that measures immunological response to HIV infection (shouldn’t this biological response not be dependent on geographic context?)?*

**Authors’ response:**

The estimation of HIV incidence by RITA depends on two key parameters : the average duration of infection (ω) and the false recent rate (ε) and their associated uncertainties, which also quantify the share of the recent among the infected and the precision of this quantification. These parameters are directly related to population levels of HIV prevalence, which determines the proportion of infected and uninfected ; and the incidence, which determines the share of the recently infected. HIV prevalence is different in different geographical contexts, that is why we say that the RITA test must be adapted to the geographical context, i.e. here to the Ivorian context. (lines 182, 185-186)

***Methods***

*I appreciate the authors specifying who is the target population for this study (i.e., not all FSW). However, the authors specify that part of the target population are FSW who could potentially benefit from PrEP. Which FSW do the authors believe would not potentially benefit from PrEP in the future, given the high prevalence of HIV among FSW?*

*Specifically, how was recruitment done for this study? Were FSW incentivized to participate?*

*Because, as the authors admit, this is a convenience sample, I do not think it makes sense to include CIs for the HIV incidence rate, which assumes the data come from a probability distribution. Could the authors please comment?*

*How is the HIV incidence calculation impacted by this being a convenience sample ? Put another way, how is the HIV incidence calculation robust to this being a convenience and not a random (or otherwise probabilistic) sample?*

**Authors’ response:**

We thank the reviewer for this comment. Indeed, our sample is not representative of the whole of FSWs. Nevertheless, in the same study, we assessed the acceptability of offering PrEP to this population. And almost all the FSWs interviewed would accept taking PrEP if it were offered to them.

The recruitment of participants for this study was made possible by the peer educator networks of two community organizations. FSW are identified by the peer educator and they work at sites that are usually visited by them for HIV prevention/screening. Almost all of the FSW work sites were visited by the peer educator. The FSWs were recruited both in prostitution sites (brothel, beaches, hotel, bar/maquis) and in the fixed clinics dedicated to sex workers of both NGOs. The recruitment involved FSWs who had never been tested for HIV or who had previously tested negative for HIV, and who were working at a sex work site at the time of the survey, in the areas targeted by the two NGOs. There was no incentive to participate in the study. All that was required was written consent to participate. (lines 160-164).

Due to the small sample size, we believe that it is important to provide a sense of the uncertainty of our estimates, in particular to see if differences between groups are relevant or not.

Considering that multivariate analysisis not possible with RITA, we believe that 95% CI constitutes a relevant indicator of the uncertainty.

We note that the assessment of HIV incidence in this key population is rare in our context, particularly in Côte d'Ivoire where the study took place. And the use of recent infection tests is a first and could provide a basis for future studies of incidence estimates in larger samples and in other populations in Côte d'Ivoire.

***Results***

*Although results are taken from convenience samples and therefore we should be cautious about directly comparing the samples, differences in the socio-demographic composition of the two samples are striking. Are there differences in the socio-demographic characteristics in the towns themselves that could explain some of the differences in the FSW populations ?*

**Authors’ response:** We thank the reviewer for this comment

There are differences in socio-demographic characteristics between the two cities. Indeed, Abidjan is the economic capital of the country, in full expansion compared to San Pedro where the level of poverty, literacy or education is lower. San Pedro was in the recent past the largest slum in West Africa and most of the FSWs sites are within the perimeters of this slum. (lines 242-246).

---------------------------------------------------------------------------------------------------------------------------

***Reviewer: 3***

*The manuscript is very well-written, and speaks to an extremely important topic for improving HIV programming. A few issues require addressing in order to strengthen conclusions and improve clarity.*

1) *Line 170: “Price of the pass” means what?*
2) *Lines 176-181: over the past several years, countries have been utilizing various assays for recent infection surveillance, with varying MDRI and FRR values. The specifics of the assay and algorithm used in this study need to be described in this section.*
3) *Lines 311-317: given that 64.7% (per Table 1) of participants reported having had an STI in the past 12 months, while 86.9% reported “always” using condoms, perhaps the authors should comment on the reliability of reported condom use.*
4) *Lines 318-321: it warrants mentioning that recent infection surveillance is a powerful tool with which Cote d’Ivoire’s national HIV/AIDS program may identify geographic areas and demographic groups within which HIV transmission is ongoing. This study is an excellent example of that, and similar such explorations more broadly would lend important insight into transmission dynamics in a high-stigma environment.*
5) *Lines 322-327: if viral load data is not integrated into the RITA, this constitutes a major limitation of the study. Without VL data, reported recent infections may include individuals who did not disclose previously known HIV+ status, who retested having been on ART, and who are therefore misclassified. The possibility of misclassification has not been discussed in this paper and needs to be addressed. Moreover, quantifying re-testers (those who test positive on recency assays, but are actually virally suppressed because of ART) would be programmatically relevant in a highly stigmatized population and environment such as this one.*

**Authors’ response:** We thank the reviewer for this relevant and important remark

1) « Price of the pass » mean the price of a single sexual encounter with a client. This clarification has been made in the text. (line 174)
2) In order to be more precise, we have completed the text : See section “Assessment of HIV incidence” for more details. (lines 185-186)
3) The point is well made. Indeed, the high percentage of STIs reported in the last twelve months contrasts with the proportion of systematic condom use. This could be the effect of temporality. Indeed, STIs were reported in the last 12 months while responses on condom use during sex were related to current use, in the week or month before the survey. Also, the presence of STIs could encourage and incite the FSW to systematically wear a condom during her sex work activity. The condom would be a means of preventing possible STIs.

We have completed the discussion as follows : FSW’ responses to the question assessing condom use might refer to ‘typical use’ as opposed to specific circumstances [ref 25]**.** Also the difference between reported STI cases and the proportion of condom use could be the effect of temporality. Indeed, STIs were reported in the last 12 months while responses on condom use during sex were related to current use, in the week or month before the survey. (lines 331-335)

4) We have included this remark in the manuscript: “Recent infection surveillance is a powerful tool with which Cote d’Ivoire’s national HIV/AIDS program may identify geographic areas and demographic groups within which HIV transmission is ongoing. More broadly, similar explorations would lend important insight into transmission dynamics in a high-stigma environment » (lines 342-346)
5) Viral load data are taken into account by RITA tests. Indeed, there is substantial evidence that a proportion of people with long-standing HIV infection are misclassified as newly infected by currently available tests for recent HIV infection. Therefore, the false recent rate of the RITA that depend on these tests can never be considered as zero. The false recent rate takes into account one or more of the following characteristics :

- Advanced infection, defined by a diagnosis of AIDS or a low CD4+ T cell count ;
- Ongoing antiretroviral treatment ;
- « Elite controllers » who have a low or undetectable viral load (*World Health Organization, editor. When and how to use assays for recent infection to estimate HIV incidence at a population level. Geneva, Switzerland: World Health Organization; 2011)*

The formula for calculating HIV incidence based on the results of a RITA therefore incorporates the false recent rate of the RITA into the incidence calculation. It is necessary to ensure that the false recent rate applied is relevant to the RITA and to the population for which the impact is estimated. This is why in our study we used the false recent rate from a sample of the Ivorian population.

A sentence was added in the discussion : « As RITA is a biological assay, classification of infections as recent or not does not rely on self-reported information. Some people with long-standing HIV infection and on treatment may be misclassified as newly infected. However, these false recent cases are taken into account, as a false recent rate is applied when estimating incidence”. (lines 348-352)
