# Supplementary material for: Incidence of HIV infection and associated factors among female sex workers in Côte d’Ivoire, results of the ANRS 12361 PrEP-CI study using recent infection assays
Source: PLoS One. 2022 Nov 17;17(11):e0271988. doi: 10.1371/journal.pone.0271988 (PMC9671321; doi:10.1371/journal.pone.0271988)
Supplement: Attachment — Submitted filename: Response_to_reviewer_PLOS ONE_2022-06-30.docx [file pone.0271988.s003.docx]

**To the editors of the Journal of the PLOS ONE**

Abidjan, June 28^th^ 2022

**Response-to-reviewers letter and revised manuscript entitled “Incidence of HIV infection and associated factors among female sex workers in Côte d’Ivoire, results of the ANRS 12361 PrEP-CI study using recent infection assays”**

Dear Editors,

This document provides point-by-point responses to the reviewers’ comments. Their comments, reproduced in *italics*, are followed by our responses and, finally by the additional text and changes to the manuscript second submitted. We thank the reviewers for their comments which have helped improve our paper.

***Reviewer : 1***

*The data availability has restricted access and is not freely available.
The manuscript needs to be edited - Table 1 and Table 2 -should state - 3 years or less and more than 3 years . No need to put more symbol as well as as state more. lines 158, 303, 308, 330, 348 need to be corrected*

**Authors’ response:**

We thank the reviewer for this comment. The data are available on Zenodo.org, with the following link <https://zenodo.org/record/5948841>. Access to the data is available on request on the website **Zenodo.org**. We mentioned this in the manuscript (line 212). In addition, we have attached in this new revision of the manuscript, the scripts and the analysis reports.

Also, we have corrected tables 1 and 2 by removing the symbol at the level of 3 years or less and more than 3 which was unnecessary. Corrections have also been made to the lines *286, 287, 297, 300.*

----------------------------------------------------------------------------------------------------------------------

***Reviewer : 2***

*The authors have been responsive to reviewer comments made by myself and the other reviewers. I am mostly satisfied, however, I still strongly believe that it is inappropriate to provide confidence intervals for the HIV incidence rates because the data they are using comes from a convenience sample (not a probability-based sample). Formulas to calculate confidence intervals assume some kind of probability-based sample. I appreciate that the authors want to communicate a sense of uncertainty in their estimates but what is the benefit if the confidence intervals themselves are wrong and therefore ultimately uninformative ? At the very least, this must be acknowledged in the limitations ; although I think it would be more appropriate to remove the confidence intervals altogether (and explain why they are not included) or do a bootstrapping technique to estimate confidence intervals****.***

**Authors’ response:** We thank the reviewer for this relevant and important remark. Indeed, confidence intervals provide for the HIV incidence rates are inappropriate because our data become from a convenience sample. In order to find a more appropriate solution, we used a bootstrapping technique to estimate our confidence intervals as suggested by the reviewer. These confidence intervals were very wide, so we decided to remove them on the differents estimated incidences (see table 2) and we have mentioned this in the limitations of the study (lines 374 – 375).

We have attached in this new revision of the manuscript, the scripts and the analysis reports for details.

---------------------------------------------------------------------------------------------------------------------------

***Reviewer : 3***

Thank you to the authors, once again, for this excellent manuscript. One of the initial questions was not addressed:
2) Lines 176-181: over the past several years, countries have been utilizing various assays for recent infection surveillance, with varying MDRI and FRR values. The specifics of the assay and algorithm used in this study need to be described in this section.

In response to this question, the authors' response was:
2)In order to be more precise, we have completed the text : See section “Assessment of HIV incidence” for more details. (lines 185-186)

However, the question was about the specifics of the recency assay utilized. The manuscript states (lines 179-182): "Then, a dried blood spot (DBS) sample was taken and transported to the laboratory of the University Hospital of Tours, France, to determine the window of infection (0.3 years) and false positive rate (13%) using a recent infection test adapted to the Ivorian context [24] and performed directly on plasma."

Are there no additional details available regarding this assay ? What is "a recent infection test adapted to the Ivorian context ?" Is it a LAg-EIA ? There are at least 10 different HIV recency assays currently and commercially available. Can the authors not shed any additional light on the assay that was utilized at University Hospital of Tours, akin to the specifics provided for the initial HIV rapid testing?

**Authors’ response:** We thank the reviewer for this relevant and important remark

We have given further details by describing the principle of carrying out this test as follows :

This recent infection test developed by Barin et al. is the Less-sensitive enzyme Immunodominant assay recent infection (EIA-RI/IDE-V3). This assay uses the enzyme immunoassay technique in a 96-well plate, based on the measurement of absorbances (OD) in one well sensitised with an equimolar peptide mixture TM (cons+D), corresponding to the immunodominant epitope of gp41 (consensus sequence envi - 1 group M and consensus sequence env - 1 subtype D), and in another well, sensitised with a V3 peptide solution (AE), corresponding to an equimolar mixture of the consensus sequences of the V3 region of gp120 of the HIV subtypes A, B, C, D and CRF01_AE. This test is an in-house test, applicable in the HIV-NRC virology laboratory, serology sector. The test can be performed on serum or plasma, as well as serum, plasma or whole blood on blotting paper or dried blood spots. The test uses a mathematical formula that combines the quantitative responses to gp41 antigens in each region to distinguish between recent and established infection.

This in-house test has been the subject of preliminary studies using sequential serum samples from HIV-infected Ivorian patients with known dates of infection (the PRECO-CI ANRS 12277 and PRIMO-CI ANRS 1220 projects) and samples from patients at different stages of the disease (the Temprano ANRS 12136 and Trivacan ANRS 1269 trials) ; which allowed to distinguish a recent infection (≤180 days) from an established infection (>180 days) with a window of infection (0.3 years) and false positive rate (13‰) for the Ivorian population studied.

## We have thus completed the paragraph “HIV screening and laboratory” in the manuscript (lines 185 - 202)
